# Potential impact of aortic stenosis diagnosis on mortality and other in-hospital complications in patients with pancreatic cancer undergoing pancreaticoduodenectomy

**DOI:** 10.1097/j.pbj.0000000000000302

**Published:** 2025-09-10

**Authors:** Adham E. Obeidat, Ratib T. Mahfouz, Parthav K. Shah, Landon A. Kozai, Mohammad R. Darweesh, Mahmoud M. Mansour, Ahmad A. Yassine, Scott K. Kuwada, Christopher H. Chang

**Affiliations:** a Department of Internal Medicine, Presbyterian Healthcare System, Albuquerque, NM 87106; b Department of Nephrology, Henry Ford Hospital, Detroit, MI 48202; c Department of Cardiology, University of Hawaii, Honolulu, HI 96813; d Department of Internal Medicine, University of Hawaii, Honolulu, HI 96813; e Department of Gastroenterology, East Tennessee State University, Johnson City, TN 37614; f Department of Gastroenterology, University of Missouri/Columbia, Columbia, MO 65212; g Department of Gastroenterology and Hepatology, University of New Mexico, Albuquerque, NM 87106; h Department of Gastroenterology, University of Hawaii, Honolulu, HI 96813

**Keywords:** aortic stenosis, pancreatic cancer, pancreaticoduodenectomy, mortality, pancreas

## Abstract

**Background:**

Patients with aortic stenosis undergoing noncardiac surgery pose a dilemma to physicians as they are at an increased risk for complications. This study aims to investigate the effect of aortic stenosis on mortality and other complications in patients with pancreatic cancer undergoing pancreaticoduodenectomy.

**Methods:**

We investigated patients with pancreatic cancer undergoing pancreaticoduodenectomy between 2016 and 2019 using the National Inpatient Sample database. The study population was divided based on the presence or absence of aortic stenosis. Multivariate logistic regression analyses were performed to determine factors associated with in-hospital mortality and other complications.

**Results:**

Of the 16,150 patients with pancreatic cancer who underwent pancreaticoduodenectomy, 165 patients were diagnosed with aortic stenosis. The mean age of patients with aortic stenosis was significantly higher. Patients with aortic stenosis had a significantly higher in-hospital mortality, occurrence of cardiac arrest, and ICU admission compared with patients without aortic stenosis. There was no difference in mechanical ventilation, hospital charges, and length of stay between the two groups.

**Conclusions:**

Aortic stenosis was found to be associated with higher in-hospital mortality and worse outcomes in patients with pancreatic cancer undergoing pancreaticoduodenectomy. Preoperative risk stratification and a multidisciplinary approach to perioperative management, among other measures, should be considered to improve outcomes.

## Introduction

Pancreatic cancer is the fourth most common cause of cancer deaths in the United States (US), with a 5-year survival rate of 8 % despite advancements in medical care.^1,2^ It is more prevalent in men and the elderly.^3^ Pancreaticoduodenectomy (PD), also known as the Whipple procedure, is a complex surgical procedure indicated for the management of both resectable and borderline resectable pancreatic cancer.^4^ It is considered a high-risk procedure due to the complex anatomy and blood supply of the area, difficult intra-abdominal dissection, and reconstruction of the digestive system. Previous reports have shown higher mortality and perioperative morbidity due to the complex nature of the procedure.^5,6^

Aortic stenosis (AS) is the most common valve disease in the elderly, and the prevalence of AS is rising among the aging population.^7^ The mortality rates depend on the severity of the AS, with the highest mortality seen in patients with symptomatic severe AS.^8^ Patients with AS are at increased risk for complications after noncardiac surgeries, including intraoperative hypotension, myocardial ischemia or infarction, heart failure, stroke, arrhythmias, and death.^9-12^ These patients require detailed preoperative assessment and risk stratification to decide which AS patients can safely undergo a noncardiac surgery and which patients require further evaluation and management beforehand.^9^

Previous studies have shown that patients undergoing PD with a history of coronary artery disease (CAD) or congestive heart failure (CHF) had almost three times the risk of developing major perioperative complications.^10^ However, to the best of our knowledge, there are no data on outcomes of patients with AS undergoing PD. In this retrospective nationwide study, we aim to evaluate the effect of AS on in-hospital mortality and other in-hospital complications in patients with pancreatic cancer undergoing PD.

## Methods

### Data source

This is a retrospective cohort study of patients who were admitted to hospitals in the United States in the period between 2016 and 2019. These data were extracted from the Healthcare Cost and Utilization Project National Inpatient Sample (NIS) database. The NIS is sponsored by the Agency for Healthcare Research Quality and is considered the largest publicly available inpatient health care database in the United States.^11^ A 20% probability sample was collected and subsequently weighted to ensure that the selected population was nationally representative. Each admission in the database was assigned one principal diagnosis, up to 40 secondary diagnoses, and 25 procedures. These variables are defined through the International Classification of Disease, 10th revision, and Clinical Modification (ICD-10-CM) codes.

### Study variables

The target population age was 18 years and older. Using ICD-10-CM codes, we were able to identify patients who had a diagnosis of pancreatic cancer and underwent PD during admission. Patient age, sex, race (White, black, Hispanic, and other), and hospital information (region and bed size) were collected and considered as baseline characteristics. Comorbidities included in the analyses were hypertension (HTN), chronic kidney disease (CKD), CHF, CAD, atrial fibrillation (AF), smoking, and obesity (defined as body mass index of more than 30). The population was divided into AS and non-AS groups based on ICD-10 codes. An intensive care unit (ICU) admission was defined as any patient who had a cardiac arrest, required vasopressor administration, or mechanical ventilation.

### Statistical analysis

Statistical analysis was performed using STATA software, version 17.0 (StataCorp, College Station, TX, USA). The characteristics of patients in the AS group and the non-AS group were described using descriptive statistics. Multivariate logistic regression analysis was performed to determine factors associated with in-hospital mortality, mechanical ventilation, cardiac arrest, vasopressor use, and ICU admission. Variables that were not statistically significant (*P*-value > 0.05) by univariate analysis were excluded from the multivariate analysis except for age, sex, and race, which were retained due to their clinical relevance. The odds ratio (OR) at 95% confidence interval (CI) was used to describe the association between study and outcome variables. Statistical significance was defined as a two-tailed *P*-value of < 0.05.

## Results

### Demographics and clinical characteristics

Of 16,150 patients carrying the diagnosis of pancreatic cancer who underwent PD during the same admission, 165 patients (1.0%) were diagnosed with concurrent AS. The mean age of patients with pancreatic cancer who underwent PD during the same admission and had a concurrent diagnosis of AS was significantly higher than patients without AS (74.5 years vs 66.4 years, *P*-value < 0.001). There was no difference in sex, race, or hospital characteristics between both groups. The prevalence of HTN, CHF, CAD, and AF was higher in the AS group. However, no difference was noted in the prevalence of CKD, smoking, or obesity between both groups (Table1).

**Table 1:** Demographic and clinical characteristics of patients in the AS group vs the non-AS group

Variable	No AS	AS	*P*-value
Age (mean, year)	66.41	74.51	<0.001
Sex (%)			0.09
Male	51.49%	66.67%	
Female	48.51%	33.33%	
Race (%)			0.56
White	72.29%	75.76%	
Black	8.45%	6.06%	
Hispanic	8.51%	3.03%	
Others	10.76%	15.15%	
Hospital region (%)			0.66
Northeast	20.49%	18.18%	
Midwest	22.24%	15.15%	
South	36.07%	45.45%	
West	21.21%	21.21%	
Hospital bed size (%)			0.71
Small	6.19%	9.09%	
Medium	18.74%	21.21%	
Large	75.07%	69.7%	
Comorbidities (%)			
HTN	60.59%	84.85%	<0.001
CKD	6.91%	15.15%	0.06
CHF	4.1%	18.18%	<0.001
CAD	15.08%	36.36%	<0.001
AF	10.38%	35.38%	<0.001
Smoking	40.1%	42.42%	0.78
Obesity	14.63%	15.38%	0.86

AS, aortic stenosis; AF, atrial fibrillation; HTN, hypertension; CKD, chronic kidney disease; CHF, congestive heart failure; CAD, coronary artery disease.

### Inpatient outcomes

#### Comparison of total hospital charges, length of stay (LOS), and in-hospital mortality

Total hospital charges were not significantly different in the AS group compared with the non-AS group ($205,231 vs $176,265, *P*-value 0.680). The mean LOS in AS group and the non-AS group was not significantly different either (12.3 days vs 11.45 days, *P*-value 0.596). On the other hand, in-hospital mortality (7.7% vs 2%, *P*-value 0.001), cardiac arrest (9.1% vs 1.0%, *P*-value < 0.001), and ICU admission (18.2% vs 6.3%, *P*-value 0.006) were significantly higher in the AS group compared with the non-AS group. There was no significant difference in the use of mechanical ventilation between the two groups (Table 2).

**Table 2:** Clinical outcomes in patients with pancreatic cancer underwent pancreaticoduodenectomy in AS vs non-AS groups

Variable	No AS	AS	*P*-value
Mean total hospital charges ($)	176,265	205,231	0.309
Mean LOS (days)	11.45	12.3	0.596
In-hospital mortality (%)	2.28%	12.12%	0.0003
Mechanical ventilation	2%	2%	0.4967
Cardiac arrest	1.03%	9.09%	<0.001
ICU admission	6.26%	18.18%	0.0065

AS, aortic stenosis; ICU, intensive care unit; LOS, length of stay.

#### In-hospital mortality

After adjusting for age, sex, race, and comorbidities, patients with AS had a significantly higher risk for in-hospital mortality compared with patients without AS (OR 3.86, CI 95% 1.18–12.63; *P*-value 0.025). The presence of CHF was independently associated with higher mortality as well. Patients with AF had a higher risk of in-hospital mortality by univariate analysis, but not by multivariate analysis. Interestingly, smoking was associated with a statistically significant decrease in the chance of in-hospital mortality in both univariate and multivariate analyses (Table 3).

**Table 3:** Univariate and Multivariate analysis of potential factors affecting the in-hospital mortality in the AS vs non-AS groups

In-hospital mortality	Univariate OR (CI 95%)	*P*-value	Multivariate OR (CI 95%)	*P*-value
AS	5.89 (1.99–17.40)	0.001	3.86 (1.18–12.63)	0.025
Age <65 years	-	-	-	-
Age ≥65 years	1.37 (0.84–2.24)	0.195	1.16 (0.69–1.93)	0.563
Male	-	-	-	-
Female	0.67 (0.42–1.07)	0.095	0.67 (0.42–1.08)	0.103
White	-	-	-	-
Non-White	1.42 (0.88–2.28)	0.144	1.5 (0.92–2.44)	0.104
HTN	1.066 (0.67–1.69)	0.785	-	-
CKD	1.78 (0.87–3.64)	0.109	-	-
CHF	3.55 (1.77–7.1)	<0.001	2.65 (1.78–3.93)	<0.001
CAD	1.46 (0.83–2.56)	0.178	-	-
AF	2.424 (1.27–3.95)	0.005	1.67 (0.86–3.23)	0.126
Smoking	0.55 (0.33–0.92)	0.023	0.56 (0.33–0.94)	0.03
Obesity	1.08 (0.57–2.01)	0.809	-	-

Variables that were not statistically significant (*P*-value < 0.05) on univariate analysis were excluded from the multivariate analysis.

AF, atrial fibrillation; AS, aortic stenosis; CI, confidence interval; CKD, chronic kidney disease; CHF, congestive heart failure; CAD, coronary artery disease; HTN, hypertension; OR, odds ratio.

#### Mechanical ventilation, cardiac arrests, and ICU admissions

AS was significantly associated with an increased chance of mechanical ventilation (OR 2.93, CI 95% 1.07–7.98; *P*-value 0.035) and cardiac arrest (OR 3.29, CI 95% 1.09–9.98; *P*-value 0.035). There was an increase in ICU admission in patients with AS; however, this was not statistically significant (OR 2.19, CI 95% 0.82–5.81; *P*-value 0.115) (Tables 4‒6).

**Table 4:** Univariate and multivariate analysis of potential factors affecting mechanical ventilation in the AS vs non-AS groups

Mechanical ventilation	Univariate OR (CI 95%)	*P*-value	Multivariate OR (CI 95%)	*P*-value
AS	4.51 (1.8–11.27)	0.001	2.93 (1.07–7.98)	0.035
Age <65 years	-	-	-	-
Age ≥65 years	1.55 (1.08–2.22)	0.015	1.28 (0.88–1.86)	0.189
Male	-	-	-	-
Female	0.93 (0.67–1.29)	0.69	1 (0.71–1.4)	0.998
White	-	-	-	-
Non-White	1.36 (0.97–1.92)	0.074	1.44 (1.01–2.05)	0.039
HTN	1 (0.72–1.39)	0.987	-	-
CKD	1.67 (1–2.8)	0.05	1.19 (0.83–2.26)	0.542
CHF	4.22 (2.59–6.86)	<0.001	2.86 (1.61–5.08)	<0.001
CAD	1.91 (1.31–2.79)	0.001	1.42 (0.91–2.23)	0.118
AF	1.99 (1.29–3.06)	0.002	1.37 (0.83–2.26)	0.215
Smoking	0.63 (0.44–0.88)	0.008	0.66 (0.46–0.93)	0.02
Obesity	1.48 (0.99–2.2)	0.053	-	-

Variables that were not statistically significant (*P*-value < 0.05) on univariate analysis were excluded from the multivariate analysis.

AF, atrial fibrillation; AS, aortic stenosis; CI, confidence interval; CKD, chronic kidney disease; CHF, congestive heart failure; CAD, coronary artery disease; HTN, hypertension; OR, odds ratio.

**Table 5:** Univariate and multivariate analysis of potential factors affecting the chance of cardiac arrest in the AS vs non-AS groups

Cardiac arrest	Univariate OR (CI 95%)	*P*-value	Multivariate OR (CI 95%)	*P*-value
AS	9.58 (2.77–33.18)	<0.001	3.29 (1.09–9.98)	0.035
Age <65 years	-	-	-	-
Age ≥65 years	2.74 (1.19–6.29)	0.017	1.42 (0.79–2.54)	0.23
Male	-	-	-	-
Female	0.466 (0.22–0.95)	0.036	0.59 (0.35–1)	0.05
White	-	-	-	-
Non-White	1.15 (0.56–2.34)	0.39	1.28 (0.77–2.13)	0.328
HTN	1.94 (0.91–4.14)	0.086	-	-
CKD	2.7 (1.11–6.55)	0.028	2.16 (1.18–3.96)	0.012
CHF	4.67 (1.9–11.45)	0.001	1.38 (0.61–3.12)	0.432
CAD	3.18 (1.59–6.35)	0.001	0.85 (0.44–1.63)	0.628
AF	4.87 (2.45–9.68)	0.001	4.84 (2.71–8.66)	<0.001
Smoking	0.84 (0.42–1.67)	0.624	-	-
Obesity	0.94 (0.36–2.43)	0.902	-	-

Variables that were not statistically significant (*P*-value < 0.05) on univariate analysis were excluded from the multivariate analysis.

AF, atrial fibrillation; AS, aortic stenosis; CI, confidence interval; CKD, chronic kidney disease; CHF, congestive heart failure; CAD, coronary artery disease; HTN, hypertension; OR, odds ratio.

**Table 6:** Univariate and multivariate analysis of potential factors affecting the chance of ICU admission in the AS vs non-AS groups

ICU admission	Univariate OR (CI 95%)	*P*-value	Multivariate OR (CI 95%)	*P*-value
AS	3.33 (1.32–8.34)	0.01	2.19 (0.82–5.81)	0.115
Age <65 years	-	-	-	-
Age ≥65 years	1.39 (1.03–1.86)	0.028	1.19 (0.87–1.63)	0.261
Male	-	-	-	-
Female	0.9 (0.68–1.2)	0.506	0.99 (0.73–1.33)	0.951
White	-	-	-	-
Non-white	1.19 (0.87–1.62)	0.269	1.28 (0.77–2.12)	0.323
HTN	1.01 (0.75–1.35)	0.92	-	-
CKD	1.65 (1.03–2.62)	0.034	1.28 (0.77–2.12)	0.323
CHF	3.59 (2.29–5.61)	<0.001	2.56 (1.52–4.3)	<0.001
CAD	1.75 (1.25–2.45)	0.001	1.29 (0.87–1.92)	0.198
AF	2 (1.38–2.89)	<0.001	1.5 (0.98–2.29)	0.061
Smoking	0.79 (0.58–1.05)	0.116	-	-
Obesity	1.39 (0.98–1.98)	0.06	-	-

Variables that were not statistically significant (*P*-value < 0.05) on univariate analysis were excluded from the multivariate analysis.

AF, atrial fibrillation; AS, aortic stenosis; CI, confidence interval; CKD, chronic kidney disease; CHF, congestive heart failure; CAD, coronary artery disease; HTN, hypertension; OR, odds ratio.

## Discussion

In this nationwide study, we found that patients with a history of AS who had pancreatic cancer and were admitted for PD had a higher in-hospital mortality than patients without AS. Patients with AS who need noncardiac surgeries present a clinical challenge. AS is a fixed obstruction of left ventricular emptying that results in left ventricular hypertrophy, poor compliance, and increased end-diastolic pressures due to alterations in the left ventricular myocardium.^12^ Therefore, these patients are more sensitive to any alterations in hemodynamics. Thus, patients with AS have a higher chance of clinical deterioration during anesthesia and surgery.^13^ Moreover, previous studies showed that patients with AS who undergo a noncardiac surgery had a higher rate of postoperative cardiovascular complications.^14-17^

Aside from hemodynamic collapse, arrhythmias can also complicate perioperative outcomes. Our study showed that patients with AS had a statistically significant higher chance of cardiac arrest, which is one of the common complications seen in patients with AS undergoing a noncardiac surgery as demonstrated by previous studies.^18,19^ Moreover, patients in the AS group had a significantly higher chance of requiring mechanical ventilation during hospitalization and a slightly increased risk of ICU admissions compared with the other group. This could be explained by the presence of greater perioperative and postoperative complication among the AS group. In addition, patients with AS might sometimes need closer hemodynamic monitoring after the surgery.

Selecting the correct and most optimal management strategy for cancer patients with AS is complex. Aortic valve replacement (AVR) is currently the only treatment option which has shown to improve hemodynamic and functional status.^20^ Previous studies have shown that patients with cancer are more often treated with conservative management rather than with AVR.^21,22^ Patients undergoing medical management usually have poor prognosis.^21^ Studies have also shown that patients who had undergone AVR before noncardiac surgery had better outcomes compared with patients who have not undergone AVR.^23^ Owing to the nature of our study, it was difficult to know which patients had already undergone AVR. However, we hypothesize that this could have resulted in poor outcomes in patients with pancreatic cancer undergoing PD. Randomized trials are needed to determine the best therapeutic strategy for cancer patients with AS (SAVR vs TAVR vs medical management). Studies are also needed to determine the optimal sequence of management: “AVR first” strategy or “surgery for malignancy first” strategy. A multidisciplinary team of surgeons, cardiologists, and oncologists can work together to determine the proper management strategy for AS patients undergoing PD.

Patients with cancer usually have symptoms that are similar to those seen in patients with AS due to various reasons such as frailty, decreased physical activity, antineoplastic medication side effects, and presence of multiple medical comorbidities. Hence, we think that symptomatic AS may be underdiagnosed in patients with cancer. A study conducted by Minamino-Muta et al found that the absence of symptoms and limited life expectancy unrelated to AS were the main reasons for choosing conservative management in patients with cancer. In these patients, surgical risk by different risk scores and echocardiographic findings had minimal effect on management strategy.^22^ As seen in our study, patients with AS undergoing PD have a high operative risk and have worse clinical outcomes. Therefore, we recommend that in patients with AS, it is important to do a detailed preoperative evaluation before undergoing PD to see if the patient would benefit from AVR before undergoing PD.

There are several limitations of this study. Owing to the nature of the NIS database, our observations reflect admissions and not individual patients. Therefore, the unit of analysis is admission. Given the inability to account for multiple admissions for a given patient in the NIS, our conclusions may be confounded by the risk of repeat hospitalization. Thus, our reported rates may be viewed as overestimates of a per-patient admission rate. Mortality rates, however, are unlikely to be affected. Undercoding or overcoding can lead to misclassification, although a large number of patients in the database strongly mitigate against substantial misclassification bias. NIS undergoes data quality assessment annually to ensure the internal validity of the data. Moreover, patients in the AS group were not classified based on the severity of stenosis, and severity can potentially affect the outcome. In addition, the number of patients who had AS was small, which limits the power of the study and probably underestimated the significance of AS on outcomes. In addition, observational studies may not be able to fully adjust for unmeasured confounding factors that might affect our estimates for the reported associations between in-hospital mortality and included covariates. Therefore, conclusions based on these observational data should be viewed as associations and not causal in nature. In addition, the number of patients who had AS was small, which limits the power of the study and probably underestimated the significance of AS on outcomes. Finally, these observations pertain to the AS population in the United States and may not be generalizable to AS populations in other countries.

As a conclusion, patients with AS had an increased hospital mortality, cardiac arrest, and ICU admissions compared with non-AS patients. Therefore, patients with concurrent diagnosis of AS and pancreatic cancer undergoing PD warrant a closer observation. A multidisciplinary approach involving oncologists, surgeons, cardiologists, and anesthesiologists should be performed to jointly develop an appropriate preoperative and postoperative evaluation and management for these patients to prevents such complications. These findings are preliminary and should be viewed with caution, as additional prospective studies are required to confirm and further explore these results.
